# Assessment on the Knowledge and Reported Practices of Women on Maternal and Child Health in Rural Sierra Leone: A Cross-Sectional Survey

**DOI:** 10.1371/journal.pone.0105936

**Published:** 2014-08-28

**Authors:** Joseph Sam Kanu, Yuan Tang, Yawen Liu

**Affiliations:** Department of Epidemiology & Biostatistics, School of Public Health, Jilin University, Changchun City, Jilin Province, P. R. China; University of New South Wales, Australia

## Abstract

**Background:**

Globally, Sierra Leone is ranked among the countries with the worst maternal and child health indicators. The mortality of women and children is significantly higher compared with other developing countries. The death of women and children can be prevented by simple cost-effective community-based interventions. The aim of this present study was to learn the knowledge levels of women on maternal and child health, and treatment-seeking and preventive behaviours in rural Sierra Leone and provide appropriate suggestions for policy makers. Moreover, the study also aimed to evaluate the effect of a husband’s involvement on health knowledge and practices of women in rural Sierra Leone.

**Methods:**

Women with at least a child of five years or below were interviewed in their households through a structured questionnaire. Characteristics of the households and of the respondents were collected and the number of correct answers given to the health knowledge and practice questions and their percentage distributions were tabulated and an overall health knowledge score was calculated.

**Results:**

The mean score of the derived overall health-related knowledge was 61.6% (maximum of 91% and a minimum of 18%) with a standard deviation of 14.7% and a median of 63.3%. Multivariable regression analyses showed education and number of pregnancies are associated with knowledge score, with significantly improved health knowledge scores amongst those who accessed higher education. There were some inappropriate practices in hygiene and sanitation. However, vaccination coverage was high with almost 100% coverage for BCG.

**Conclusions:**

Based on the findings of this study, women’s knowledge on maternal and child health care are inadequate in rural Sierra Leone. Health promotion activities focusing on prevention of diarrhoea, malaria and pneumonia, improvement in health-related knowledge on pregnancy, delivery, neonatal care and environmental sanitation would be invaluable.

## Introduction

The death of a woman while pregnant or within 42 days of termination of pregnancy, irrespective of the duration and site of the pregnancy, from any cause related to or aggravated by the pregnancy or its management but not from accidental or incidental causes i.e., maternal mortality (WHO Statistics) [1]–has been a priority area for the global health and development community at least since the Nairobi Safe Motherhood Conference in 1987 [2]. Neonatal mortality, infant mortality, and child mortality in general are still at unacceptably high levels despite the efforts invested in decreasing these mortalities in sub Saharan Africa and Southern Asia [3]. Deaths of children under the ages of five years are increasingly concentrated in these areas [3].

Sierra Leone has one of the highest child and maternal mortality rates in the world, and despite recent progress, it is not on track to reach the 2015 MDGs for MDG 4 (reducing child mortality rates) and MDG 5 (improving maternal health) unless rapid acceleration takes place [4]. The mortality rate of under-five children remains at 140 deaths per 1,000 live births and of infants at 89 deaths per 1,000 live births [5]. The maternal mortality is at 857 maternal deaths for every 100,000 live births in Sierra Leone [5].

Several major causes accounting for the mortality and morbidity of reproductive and sexual diseases have been identified. Obstetric haemorrhage is the leading cause of maternal mortality worldwide, which accounts for an estimated 127 000 maternal deaths annually. Postpartum haemorrhage is the most common type of obstetric haemorrhage, causing up to 13–44% of all maternal deaths in developing countries [6]–[8]. Pre-eclampsia and eclampsia are two major health problems in developing countries [9], [10]. In rural communities, there are few or no reliable health care institutions to take care of women during child delivery, because most fairly equipped hospitals are concentrated in urban areas, which makes pregnant women to rely on Traditional Birth Attendants (TBA), who do not possess the necessary skills to carry out safe child delivery, particularly when there are complications. Infection is likely to develop after an abortion or childbirth and is a major cause of death. Sepsis that is not related to unsafe abortion accounts for up to 15% of maternal deaths in developing countries. The majority of unsafe abortions take place in developing countries [5], [9]–[12]. After pregnancy-related causes of death, sexually transmitted infections are the second most important causes of healthy life lost in women [13]. Many sexually transmitted infections affect the outcome of pregnancy and some are passed to unborn and newborn babies [13]–[16]. Preterm birth is the leading cause of neonatal mortality in both developed and developing countries, accounting for an estimated 24% of neonatal deaths [9], [17]–[20].

Pneumonia is the leading cause of death in children under five years old, killing about 1.6 million children annually and accounting for 18% of all deaths of children worldwide [21]–[23]. Diarrhoeal disease is the second leading cause of death and a leading cause of malnutrition in children under five years old, killing more than 1.3 million children every year [24], [25]. Every 45 seconds a child dies of malaria in Africa. In 2008, there were 247 million cases of malaria and nearly one million deaths – mostly children in Africa [26]. Other infections such as neonatal sepsis are also well documented as causes of death in neonates and infants [21], [27]–[29]. An estimated 164 000 people died from measles in 2008 – mostly children under the age of five [30].

Available knowledge and tools are effective in reducing maternal and newborn suffering and death [31]. Experience has shown these interventions are affordable and can be effectively delivered even in the poorest countries [31]. However, only when these available knowledge and tools are accessible, will they help the suffering people [31]–[33]. Health promotion programmes worldwide have long been premised on the idea that providing knowledge about causes of ill health and choices available will go a long way towards promoting a change in an individual’s behaviour towards more beneficial health seeking behaviour. The way in which women reach the decisions they take can have a profound effect on child morbidity and mortality and is therefore worthy of continued study [34].

In public health today, there are consistent and powerful findings revealing the strong association between mothers' education and child mortality [35]–[38]. The causal link between health outcomes and education is reported in many surveys [41]–[44]. Every one year increment in education level is associated with a 7–9% reduction in mortality of children less than 5 years old [39]. The mortality rate of children whose mothers have above 7-year schooling is 58% lower than those whose mothers have no education [40]. Other studies also share this finding; with a causal link been established between a range of health outcomes and education [41]–[44].

Accessibility to health interventions, especially among the poorest people in the world requires the improvement of poor-organized health systems,–typically characterised by insufficient numbers of health workers, poorly functioning supply chains, and low-quality care [45]. The common barriers to health services and interventions in the poorest settings include lack of financial support, unhealthy lifestyle, and cultural background [45]. These limitations result in poor delivery of health promotion messages to women during antenatal visits, vaccination and treatment of their children. In Sierra Leone, the introduction of the free health care initiative (FHCI) makes it possible for more women and children have easier access to health care. With the limited number of health workers, the heavy workload results in health workers having little time for counseling and passing on health promotion messages that are mainly delivered at the time of clinic visits.

Although the high maternal and child mortality rates in Sierra Leone are well documented in the international literature, far less is known about women's knowledge of maternal and child health in this setting.

The main aim of this study was to assess the knowledge and practices of women on maternal and child health in rural Sierra Leone. The study also sought to identify impact factor(s) of knowledge and practices and investigate the effect of the husbands’ involvement on maternal and child health. Direct observations were recorded on households and suggestion and advices on health improvement were given.

## Methodology

### Ethics statement

This study was approved by the Institutional Review Board of School of Public Health, Jilin University and by the Ministry of Health and Sanitation in Sierra Leone. Signed informed consents were obtained from all participants before the interviews.

### Study location and participants

The study was carried out in a rural area of Sierra Leone. Five villages (clusters) in the Bombali district of northern Sierra Leone were selected randomly from the list of towns/villages in the province. From each village, households with at least a woman with a child under five years old were identified. For households having more than one woman that met the inclusion criteria, only one woman was selected by simple random sampling and interviewed. As a result, a total of 244 mothers in 244 different households were selected and interviewed.

### Inclusion criteria

1. All mothers of 5-year-old children and below were included in the study.
2. All women who have given birth in the past 5 years, regardless of whether the child was still alive or not were included in the study.

### Exclusion criteria

1. Women of childbearing age who do not fulfill the inclusion criteria above were excluded from the study.
2. Women who were pregnant at the time of the study but had not given birth before and those without at least a child five years or younger, were not included in the study.

### Data collection

The data collection was done from the 1^st^ to the 16^th^ of march, 2012 using a fully structured questionnaire, developed from an earlier questionnaire by Rebecca King, Vera Mann, and Peter D. Bone, in a similar study in Guinea Bissau [46]; and all interviews were carried out at the participants’ homes after making signed informed consents. For those who were illiterate, informed consent was represented by thumb printing. The consent form was read and translated in languages the respondents understood (mostly in krio, temne and limba – all respondents understood at least one of these local languages, and all interviewers were able to communicate with the participants in these languages). Participants who reported loss of child or stillbirth were comforted by a counselor even though there were no signs of emotional distress in these participants.

The questionnaire was organized into eight sections: participant’s identification, household information, pregnancy and antenatal care, health knowledge, treatment-seeking behaviour, vaccination status, involvement of husband in maternal and child care, and household observation (key variables collected in each section are presented in Table 1). Interviews were done in local languages (krio, limba, and temne) at the participants’ homes. It was mandatory that interviews occur at home as direct observations on the participants’ dwelling places were part of the research. Interviews were done by five personnel who were well trained on the use of the questionnaire before data collection. Sections one to seven were completed by each participant whilst section eight was done by interviewer making direct observation (inspection of the household sanitary condition using checklist included in the questionnaire - see key variables collected under section 8 in Table 1). In total, 22 questions were included in deriving the heath knowledge scores with each question contributing a maximum of only one point. Questions with more than one correct answer were weighed by dividing the number of correct answers given by the total number of correct answers. The questionnaire was tested in one other village (which was excluded in the main study) and amendments made before the questionnaire was used in the main study. Responses were collected, organized and analyzed.

**Table 1:** Key Variables collected in each section.

Section	Key variables collected
1. Participant’s identification	Name of town/village; age, occupation, level of education and ethnicity.
2. Household (HH) information	Number of people in the household (including children and adults); number of women of child-bearing age (15−>45yrs); number of children under 5 years; number of living rooms in the household; number of bed nets in the household; and husband/spouse’s number of wives.
3. Pregnancy and antenatal care	Number of pregnancies, stillbirths, children, and antenatal care received in the last pregnancy; place of antenatal care and delivery; who attended delivery; care of the umbilical cord at the time of delivery; time of first breast feed; knowledge on danger signs of pregnancy; and experience of complications.
4. Health knowledge	Knowledge on diarrhoea prevention and management; knowledge on oral rehydration salts (ORS) preparation and use; knowledge on fever management; malaria prevention and management; knowledge on pneumonia, measles, and HIV/AIDS.
5. Accessing health care	Independence of the woman in making decision for seeking treatment; factors preventing women from getting medical advice or treatment; first port of call when a child is seriously sick; place of buying medicines; and length of keeping medicines at home.
6. Vaccinations coverage	Completion of vaccination (BCG, DPT, OPV, and measles); and place of vaccination.
7. Child’s father/husband’s involvement in maternal and child care	Husband’s occupation; husband’s number of wives and children; roles played by husband towards maternal child health (1 = decide when and where to take the child for treatment when sick, 2 = provide money for treatment& medicine, 3 = remind of clinic/hospital visits for vaccination, 4 = take the child to hospital when the mother is unable to do so); and rating of husband’s commitment to maternal and child/children’s health.
8. HH observation	Condition of storage of drinking water; presence of bed nets in the HH; condition of the place for food preparation; presence of latrine and its hygiene condition; types and condition of storage of medicine at home; source of drinking water.

### Analysis of Data

The data was collected on forms (questionnaires), and all forms were screened, cross-checked and package for further processing and safe keeping. The collected information was first double entered into the Epidata 3.1 data base using necessary checks (to prevent entering of values outside the range of a particular question), and was then exported to other statistical softwares (Excel & SPSS), screened again before analyses done. Both Excel and SPSS were used for data analyses. This process was managed by the first and second authors of this manuscript.

Households and demographic characteristics of the respondents were tabulated and analyzed. Descriptive statistics for continuous variables such as mean, standard deviation, median, and range and for categorical variables the numbers and percentages were calculated. The number of correct answers given to the health knowledge questions and their percentage distribution were tabulated by items. An overall health knowledge score for each participant was derived by summing the total number of correct responses given to the health knowledge questions in the questionnaire. Using the derived health knowledge score as dependent variable, factors affecting health knowledge scores were investigated using simple and multiple linear regression analyses models.

## Results

Participants’ demographic characteristics are presented in Table 2. Out of the 244 participants interviewed, 32 (13.1%) were from Gbankan town, 70 (28.7%) were from Kabombeh town, 28 (11.5%) were from Robureh, 59 (24.2%) were from Katherie, and 55 (22.5%) were from Robuya town. Respondents were mainly farmers 165 (67.6%) and traders 43 (17.6%) with 21 (8.6%) being students at the time of the interview. More than half of the interviewed women 135 (55.3%) had never attended school and thus no form of formal education; with only 3 (1.2%) attaining tertiary education.

**Table 2:** Participants’ characteristics (demographics).

Category	Subcategory	Number	%
Ethnicity	Fulla	3	1.2
	Korankoh	1	0.4
	Limba	161	66.0
	Mende	2	0.8
	Susu	1	0.4
	Temne	76	31.1
Age (yrs)	Below 20	45	18.4
	20–30	130	53.3
	31–40	60	24.6
	Over 40	9	3.7
Occupation	Farmer	165	67.6
	Hair dresser	1	0.4
	House wife	10	4.1
	Miner	1	0.4
	Student	21	8.6
	Tailor	2	0.8
	Teacher	1	0.4
	Trader	43	17.6
Education	None	135	55.3
	Primary	55	22.5
	Secondary	51	20.9
	Tertiary	3	1.2
Total		244	100.0

Table 3 and 4 give summary of the household characteristics. The average number of people per household was 9 with a minimum of 3, maximum of 25 people and a standard deviation of 4.6. On average, there were 2 females of childbearing age and a similar average of children under five years of age. The average number of living rooms in each household was 2. An average of 4 people shared a living room with an average of 2.2 bed nets in each household. Most of these bed nets 165 (67.6%) never required retreatment as they were long-lasting insecticide-treated bed nets. Direct observations made in the households by the interviewers revealed that 215 (88.1%) of the households had bed nets in the place where the youngest child sleeps. Nearly all of the observed households 230 (94.3%) had no form of well defined place for hand washing; and of the 14 (5.7%) with place for hand washing, only one household had soap for hand washing. Only 15 (6.1%) of the observed households were without latrine facilities. In more than half of the households, that is, 141 (57.8%), the main source of drinking water is from tap water, 63 (25.8%) from open wells and 40 (16.4%) from hand operated wells.

**Table 3:** Household Information (Responses of Interviewee).

Responses of Interviewee	Min.	Max.	Mean	Median	S.D
No. of people in HH*	3	25	9.1	8.00	4.6
No. of females of child-bearing age**	1	7	2.0	2.00	1.1
No. of children under 5 yrs	1	7	2.4	2.00	1.3
No. of living rooms in HH	1	9	2.4	2.00	1.3
No. of people per room	2	11	4.4	4.00	1.6
No. of bed nets in the HH***	0	6	2.2	2.00	1.2

*HH- Household.

**Child-Bearing age = 15 to 45 years of age.

***Out of the 244 participants, 5 (2.0%) responded that the bed nets did not need impregnation at the time of the study, 2 (0.8%) responded that the bed nets were treated within the last six months, 165 (67.6%) said the nets were never treated, 61 (25.0%) didn’t know whether the nets were treated or not; whilst 11 (4.5%) had no bed nets.^.^

**Table 4:** Household information (interviewer’s direct observation).

Category	Subcategory	Number	*%*
Bed nets at youngest child’s sleeping place	Yes	215	88.1
	No	29	11.9
Water storage facility	Clean	176	72.1
	Covered	202	82.8
	Dedicated ladle or cup to remove water that is not used to drink from directly	3	1.2
	Ladle or cup to remove water that is use to drink from directly	169	69.3
	others	17	7.0
Have a place for hand washing	Yes	14	5.7
	No	230	94.3
Soap present in hand washing facility?	Yes	1	7.1
	No	13	92.9
Clean latrine	Yes	80	32.8
	No	164	67.2
Latrine covered with lid	Yes	109	44.7
	No	135	55.3
Latrine with water for cleaning	Yes	4	1.6
	No	240	98.4
Latrine with brush for cleaning	Yes	4	1.6
	No	240	98.4
Latrine with place for hand washing close by	Yes	167	68.4
	No	77	31.6
Latrine condition not applicable (no latrine)	Yes	15	6.1
	No	229	93.9
Medicine at home	Good storage of medicine at home	74	30.3
	Fair storage of medicine at home	4	1.6
	Poor storage of medicine at home	48	19.7
	No medicine at home	118	48.4
Main Source of drinking water	Tap water	141	57.8
	Open well water	63	25.8
	Hand operated well water	40	16.4

Tables 5 and 6 summarize the respondents’ pregnancies and antenatal care practices. High birth rates were recorded in most of the participants with an average of 3.8 total pregnancies, a mean of 1.7 pregnancies in the last five years, and an average of 0.3 stillbirths. Participants had an average of 3 children at the time of the study. Respondents reported an average of 5.8 antenatal care (ANC) visits with most women attending health clinics 231 (94.7%) in their last pregnancies. Most participants 155 (63.5%) could not identify any danger sign that necessitates compulsory institutional (clinic or hospital) deliveries. Non-institutional deliveries were reported by 80 (32.8%) at home and 50 (20.5%) at TBAs’ places, with only 114 (46.8%) deliveries occurring in heath institutions [101 (41.4%) in clinics and 13 (5.3%) in hospitals]. In 42.2% of participants, deliveries were attended by TBAs, 40.6% attended by nurse/midwife, only 7.4% attended by medical doctors and 8.2% attended by relatives and friends. Excluding TBAs as formal skilled health personnel, in this study, only 48.0% of respondents had supervised delivery by skilled health personnel in their last pregnancy [medical doctors 18 (7.4%) and nurse/midwives 99 (40.6)]. Razor blade 138 (56.6%) and scissors 52 (21.3%) were reported to be used for cutting the umbilical cord in the last pregnancies. Immediate breast feeding 162 (66.4%) was reported for the last live delivered babies. Most respondents 173 (70.9%) gave the correct answer that at the age of six months a child can be given other food in addition to breast milk.

**Table 5:** Pregnancy and antenatal care (continuous variables).

Respondents’ responses	Min.	Max.	Mean	Median	S.D
Total no. of pregnancies (stillbirth/miscarriage/not-to-term)	1	13	3.8	3.0	2.5
Total no. of pregnancies in the last 5 years	1	3	1.7	2.0	0.6
Total no. of stillbirths/miscarriage	0	6	0.3	0.0	0.8
No. of full term pregnancies	1	11	3.5	3.0	2.2
No. of children	1	9	3.1	2.0	1.9
No. of antenatal care received in last pregnancy	0	9	5.6	6.0	1.7

**Table 6:** Pregnancy and antenatal care (categorical variables).

Category	Subcategory	Number	%
Place of antenatal care	Clinic	231	94.7
	Hospital	11	4.5
	No. antennal care visits	2	0.8
Place of delivery	At home	80	32.8
	TBA’s place	50	20.5
	Clinic	101	41.4
	Hospital	13	5.3
Who attended the delivery	TBA	103	42.2
	Nurse/midwife	99	40.6
	Doctor	18	7.4
	Relatives/friends	20	8.2
	No one (self delivered)	4	1.6
Umbilical cord cutting instrument	Razor blade	138	56.6
	Scissor	52	21.3
	Others/don’t know	54	22.1
Sterilized instrument for cord cutting	Yes	149	61.1
	Don’t know	95	38.9
How long before first breast feeding	Immediately	162	66.4
	Hours	59	24.2
	Days	22	9.0
	Others	1	0.4
Give child other food at age 6 months	Correct answers	173	70.9
	Wrong answers	71	29.1
Danger pregnancy signs	Can’t identify any danger sign	155	63.5

Responses to health knowledge are presented in Table 7. Only 107 (43.9%) of participants knew that a child with diarrhoea needs to be given more fluids to drink than usual. Although almost all respondents 243 (99.6%) knew ORS (oral rehydration salt), only 39 (19.1%) correctly explained how to prepare ORS solution from sugar, salt and water when the readymade ORS is not available. Sixty-four (26.2%) of the total respondents could not give any correct answer on the ways to prevent diarrhoea and only 29 (11.9%) were able to give all four correct answers on the prevention of diarrhoea included in the questionnaire. Only 43 (17.6%) respondents knew that a baby under 6 months with diarrhoea need to be given breast milk followed by ORS if necessary; which revealed that the overall knowledge of the respondents on the management of diarrhoea was poor. The respondents’ knowledge on fever management in children was sufficient with 209 (85.7%) knowing that a child with fever needs to be dressed lightly and cooled with damp cloth. Knowledge on malaria was poor. Although 244 (100%) of respondents had heard of malaria and 229 (93.9%) stated that people became infected with malaria from mosquito bite; only 1 (0.4%) could give all four preventive means for malaria and 17 (7.0%) could not give any preventive method for malaria. Only 100 (41.0%) of respondents knew that chloroquine should be given to a child with malaria for at least 3 days even if the child gets better. Two hundred and twenty-three - 223 (91.4%) of respondents heard of pneumonia, but up to 80 (32.8%) could not give any correct sign of pneumonia, whilst only 31 (12.7%) gave all the three signs of pneumonia as included in the questionnaire. Similarly, 109 (44.7%) knew that antibiotics are used for treatment of pneumonia. Only 72 (29.5%) knew that the best way to prevent measles was by vaccination. Assessment of the participants’ knowledge on HIV/AIDS revealed that 239 (98.1%) heard of HIV/AIDS but only 29 (11.9%) knew all the three major ways of preventing HIV infection; and only 37 (15.3%) knew that HIV/AIDS cannot be completely cured.

**Table 7:** Health knowledge*.

Category	Subcategory	Number	%
Items of healthknowledgerequired (totalnumbers ofpossible correctanswer)	Number who said to give moredrink to a child with diarrhoea	107	43.9
	Number who knew of ORS(oral rehydration salt)	243	99.6
	Number who claimed knewthe use of ORS	238	97.5
	Number who specified ORS use	224	94.1
	Number who claimed can prepareORS from salt, sugar & water	204	83.6
	Number who gave correct answer to the preparation of ORS	39	19.1
	Number who said to give breast milk followed byORS if necessary to a baby under 6 months withdiarrhoea	43	17.6
	Number who said to dress lightly and cool childwith damp cloth if child is sick with fever	209	85.7
Prevention ofdiarrhoea -number of correctanswersgiven (4)	0 (no correct answer)	64	26.2
	1 (one correct answer)	16	6.6
	2 (two correct answers)	37	15.2
	3 (three correct answers)	98	40.2
	4 (four correct answers)	29	11.9
	Number who heard about malaria	244	100.0
	Number who said people become infected withmalaria from mosquito bite	229	93.9
Number ofcorrect answersgiven to avoidmalaria (4)	0 (no correct answer)	17	7.0
	1 (one correct answer)	70	28.7
	2 (two correct answers)	74	30.3
	3 (three correct answers)	82	33.6
	4 (four correct answers)	1	0.4
Chloroquine use	Number who said to give chloroquine to a childwith malaria at least for 3 days even if the childgets better	100	41.0
Heard ofpneumonia	Number who heard about pneumonia	223	91.4
Number of correctanswersgiven for signs ofpneumonia(3)	0 (no correct answer)	80	32.8
	1 (one correct answer)	59	24.2
	2 (two correct answers)	74	30.3
	3 (three correct answers)	31	12.7
Pneumoniatreatment	Number who said that antibiotics aretreatment for pneumonia	109	44.7
Best pneumoniapreventionmode	Number who said vaccine is the correct way toavoid measles	72	29.5
Knowledge onHIV/AIDS	Number who heard about HIV/AIDS	239	98.1
Number of correctanswersgiven for how cansomeoneget HIV/AIDS (2)	0 (no correct answer)	70	28.7
	1 (one correct answer)	120	49.2
	2 (two correct answers)	54	22.1
Number of correctanswersgiven for how cansomeone preventHIV/AIDS	0 (no correct answer)	68	27.9
	1 (one correct answer)	26	10.7
	2 (two correct answers)	121	49.6
	3 (three correct answers)	29	11.9
Treatment ofHIV/AIDS	Number who said HIV/AIDS cannot becompletely cured	37	15.3

*Health Knowledge score calculated from the various health knowledge questions with each question contributing a maximum of one point. Expressed in percentages, the results gave a minimum of 18%, maximum of 91%, with mean of 61.6, median 63.3 and standard deviation 14.7.

The derived overall health knowledge revealed a mean score of 61.6% (maximum of 91% and a minimum of 18%) with a standard deviation of 14.7% and a median of 63.3%.

A Simple linear regression analyses (Tables 8 and 9) revealed people with higher levels of education achieved substantially higher scores (p<0.05) with a 2.7% increase in score with each unit increase in educational level. This was also true in the multiple regression models. Total number of pregnancy was also found to significantly influence health knowledge score (p = 0.021) but this was not statistically significant in the multiple regression models when the total number of children each participant had were included at the same time using step wise. Of statistical significance in the multiple regression model using stepwise were educational level and total number of pregnancies, and the formula was: {y_score_ = 54.3+3.8x_education_ +1.2x_no. of pregnancy_}; with both education and total number of pregnancies having p-values less than 0.05. Also investigated was the linear regression relationship between the husband’s roles scores and health knowledge score of the respondents but this was not statistically significant in both the simple and multiple regression models.

**Table 8:** Results of Linear regressions for health knowledge score (univariable model).

Variable (unit)	Change in score^a^	95% *CL*		*P*-value
Age (years)	0.6	−1.9	3.0	0.653
Husband’s score^b^	4.7	−1.7	11.1	0.148
Total no. of pregnancy	0.9	0.1	1.6	0.023
No. of children	1.2	0.3	2.2	0.013
Education	2.7	0.5	4.9	0.015

a Change in the average health knowledge score for a unit increase in the continuous variables or being in a category versus the baseline category for categorical variables.

b Husbands’ overall score out of the total roles of the husband included in the questionnaire.

**Table 9:** Results of Linear regressions for health knowledge score (multivariable model).

Variable (unit)	Change in score^a^	95% *CL*		*P*-value
Total no. of pregnancy	1.2	0.5	2.0	0.001
Education	3.8	1.6	6.0	0.001

a Change in the average health knowledge score for a unit increase in the continuous variables or being in a category versus the baseline category for categorical variables.

Accessing health care and treatment seeking behaviours of participants are summarized in Table 10. In this study 81.0% of the respondents reported they can make independent decision for a child’s treatment when seriously ill. Still considered as big problems by women in this study were getting money for needed treatment 49 (87.3%), distance to health facility 155 (63.5%), taking transport 151 (61.9%), only a male health provider present at the health facility 87 (35.7%), absence of care provider at the clinic or the clinic closed at the time of health need 138 (56.6%), the clinic unable to cure the patient 226 (92.6%), and the clinic not having the appropriate medicines 237 (97.1%). Almost half of the respondents 121 (49.6%) reported that when a child is seriously ill, their first port of call for help is their relatives, 40 (16.4%) clinic, 62 (25.4%) hospital, and up to 5 (2.0%) seek help first from traditional healers. Places of buying medicines vary greatly among respondents with 113 (46.3%) buying medicines from local clinics or hospitals, 73 (29.9%) from local persons, and 53 (21.7%) from pharmacies or drug stores; and respondents taking on average 46.9 minutes to reach these places of buying medicines.

**Table 10:** Accessing health care and treatment seeking behaviour↑.

Category	Subcategory	Number	%
Woman making decision for child’streatment when seriously ill	Yes	200	82.0
	No	25	10.2
	Depends	19	7.8
Number who said it is a bigproblem to the followingquestionnaire items:	Knowing where to go- a bigproblem	14	5.7
	Getting permission to go- a bigproblem	49	20.1
	Getting money for needed treatment-a big problem	213	87.3
	Distance to health facility- a bigproblem	155	63.5
	Taking transport- a big problem	151	61.9
	Going alone- a big problem	42	17.2
	There may only be a male healthprovider- a big problem	87	35.7
	There may not be anyone at the clinicor clinic closed- a big problem	138	56.6
	The clinic will not be able to cureyou-A big problem	226	92.6
	The clinic will not have appropriatemedicines- a big problem	237	97.1
Person from whom help is sought ifchild is seriously ill	Another parent	6	2.5
	Relative	121	49.6
	Traditional healer	5	2.0
	Matrona	2	0.8
	Clinic	40	16.4
	Hospital	62	25.4
	Others	8	3.3
If you need medicines for yourchildren, where do you normallygo to buy them?	Local person (“peppeh doctor”)	73	29.9
	Relative	1	0.4
	Pharmacy/drug store	53	21.7
	Local clinic/hospital	113	46.3
	Patent medicine shop	1	0.4
	Others	3	1.2
How long do you normally keepmedicine at home?	Can’t exactly tell	145	59.4
	Just one month	89	36.5
	More than one year	2	0.8
	Others	8	3.3

↑ Summary of the approximate time to source of medicine (Minutes) revealed a minimum and maximum of 1 and 165 minutes respectively; with mean 46.9, median 15.0, and standard deviation 49.4.

Vaccination coverage was summarized in Table 11. One hundred and ninety-nine - 199 (81.6%) of the interviewed women presented the vaccination cards for their youngest child that was alive whilst 45 (18.4%) could not; and 234 (95.9%) of these vaccinations occurred in regional clinics, 5 (2.0%) in hospitals and 5 (2.0%) in non-governmental organization (NGO) clinics. Vaccination coverage was high for the first vaccine BCG and decreased as we moved from the first to the last (measles) vaccination.

**Table 11:** Vaccination coverage*.

Completion of vaccination (*n* = 244–45 = 199)	Overall %**	Completed vaccination***		Failed to vaccinate****		Not yet due	
	%	No.	%	No.	%	No.	%
BCG≥1 week	100.0	199	100.0	0	0.0	0	0.0
OPV1≥6 weeks	98.5	196	98.5	3	1.5	0	0.0
OPV2≥10weeks	95.2	177	88.9	9	4.5	13	6.5
OPV3≥14 weeks	95.2	160	80.4	8	4.0	31	15.6
DPT1≥6 weeks	98.5	196	98.5	3	1.5	0.0	0.0
DPT2≥10 weeks	95.2	177	88.9	9	4.5	13	6.5
DPT3≥14 weeks	95.2	160	80.4	8	4.0	31	15.6
Measles≥9 months	88.6	117	58.8	15	7.5	67	33.7

*Out of the 244 participants, 199 (81.6%) had the vaccination cards for their youngest children that were alive, whilst 15 (18.4%) did not have; 234 (95.9%) received most of their vaccinations in regional clinics, 5 (2.0%) hospitals, and 5 (2.0%) from Non-Governmental Organization (NGO) clinics.

**Actual vaccination coverage calculated only for children with vaccination cards and were due for the vaccination.

***Vaccination coverage calculated for all children with vaccination cards irrespective of whether they were due for the vaccination or not.

****Failure of vaccination calculated for children with vaccination cards irrespective of whether they were due for the vaccination or not.

Table 12 summarizes the husband’s characteristics and role in maternal and child health care. Seventy-seven - 77 (31.6%) of the husbands were not staying with the participants at the time of the study. Four major roles expected to be played by husbands towards maternal and child health were included in the questionnaire. These roles include: decide when and where to take the child for treatment when sick, provide money for treatment and medicine, remind partner of clinic/hospital visits for vaccination, and take the child to hospital when partner is unable to do so. Of these, 31 (12.7%) of husbands played no role, 68 (27.9%) played only one role, 67 (27.5%) two roles, 23.8% three roles and only 20 (8.2%) contributed all the four roles investigated. On average each husband had 4 children (Minimum 1, maximum 20); 149 (61.1%) had only one wife, 82 (33.6%) had two wives, 10 (4.1%) had three wives whilst 3 (1.2%) had four wives. Only 48 (19.7%) of the respondents rated their husbands’ commitment to their health and their children’s health care as very good, and 8 (3.3%) as very poor.

**Table 12:** Summary of husband’s characteristics and role in maternal and child health care^a^.

Category	Subcategory	Number	%
Husband’s occupation	Farmer	144	59.0
	Student	29	11.9
	Builder/messenger	17	7.0
	Trader	12	4.9
	Teacher	8	3.3
	Others	34	13.9
Respondent and child/children’s father stayingtogether	Yes	167	68.4
	No	77	31.6
Number of roles played by husband inmaternal & Child health (4)^b^	0 (no role)	31	12.7
	1 (one role)	68	27.9
	2 (two roles)	67	27.5
	3 (three roles)	58	23.8
	4 (four roles)	20	8.2
Rating of husband’s commitment to respondent’sand child/children’s health care	Very good	48	19.7
	Good	101	41.4
	Fair	66	27.0
	Poor	21	8.6
	Very poor	8	3.3
Husband’s number of wives	1 (one) wife	149	61.1
	2 (two) wives	82	33.6
	3 (three) wives	10	4.1
	4 (four) wives	3	1.2

a Summary of the husbands’ number of children revealed a minimum and maximum of 1 and 20 children respectively; with mean 4.4, median 4.0, and standard deviation 3.9.

b There are four (4) main roles included in the questionnaire: Decide when and where to take the child for treatment when sick; provide money for treatment and medicine; remind partner of clinic/hospital visits for vaccination; and take the child to hospital when partner is unable to do so.

## Discussions

The sample in the study were mainly limba 161 (66.0%) and temne 76 (31.1%). Although the combined age groups 20–30 and 31–40 years of age formed the largest proportions of respondents 210 (77.9%), as much as 45 (18.4%) of the mothers interviewed were below the age of 20 years. Our finding that nearly 20% mothers were under 20 years old was in accordance with the UNFPA consultancy report on the determinants and consequences of teenage pregnancy and motherhood in Sierra Leone [47]. Most of the respondents were poor subsistence farmers 165 (67.6%), lacking formal education and 135 (55.3%) of them never attended school, putting them at disadvantage for health and other social amenities in society.

According to our study, there were on average 9 people living together per household. Within this household there was an average of 2.4 living spaces/rooms where people reside and 4.4 people sharing each room/living space. This overcrowding phenomena results in a diminished quality of life due to increased physical contact, lack of sleep, lack of privacy, poor hygiene practices and an inability to care adequately for sick household members [48]. Up to 29 (11.9%) of respondents lack bed nets in the places where the youngest child sleeps. Overall sanitary conditions were poor with 230 (94.3%) households lacking place for hand washing. These poor sanitary conditions are thus associated with outbreaks and spread of cholera and other infectious diseases.

Based on a study conducted by researchers from John Hopkins University, the interval of three to five years for childbirth is important for the health of both mother and child [49]. The most powerful impact factor for child survival is birth spacing [50]. However, according to our study, there was an average of four children in one household and 2 children were under five, which suggested that the birth interval was less than three years and resulted in poor health of mothers and child survival. It was estimated that birth control could avoid 100,000 mothers' death and death of one out of every five babies [51]. Sixty-three and half percent of the respondents could not identify any sign that indicates hospital/clinic delivery, and 32.8% of respondents admitted home deliveries. However, high numbers of antenatal care were received by respondents in their last pregnancies and 66.4% admitted immediate breastfeeding after delivery.

In rural settings, many factors can influence a woman’s access to health care facilities and treatment seeking behaviours. Despite the free healthcare initiative introduced for pregnant, lactating mothers and children under five years by the government of Sierra Leone in April 2010 (excluding women from the explicit costs for consultations, medicines and other hospital charges), there are still implicit costs or constrains that act as barriers to healthcare access. Lacking of financial support and distance to health facilities were still barriers to prompt and early treatment. Inability of healthcare facilities to provide the appropriate care and medicines and these institutions being closed at some times of need were reported as big problems by respondents in this study. Treatment seeking behaviour was also found to be inappropriate with 121 (49.6%) of respondents first contacting relatives for treatment when a child is seriously ill compared to only 40 (16.4%) who immediately take the child to the clinic; and 73 (29.9%) buy medicines from local persons (who are usually with substandard drugs and lacking knowledge of their use).

In contrast to the 2008 demographic and health Survey [4], vaccination coverage was reported in this study to be 100% coverage for BCG. However, consistent with similar studies [36], [46], vaccination coverage decreased as we moved down from the first vaccination BCG to the last, i.e., measles.

The health knowledge of women on maternal and child health varies widely as investigated in this study with calculated minimum score as low as 18% and as high as 91% (mean 61.6%, median 63.3% and standard deviation of 14.7). In accordance with other studies, our study showed that health knowledge scores improve significantly amongst those who have education [36], [41]–[44], [46], [52], [53], especially secondary school (high school) and higher. It was also found in our study that the overall health score was higher among women who were multiparous irrespective of their level of education. This might be due to repeated health education messages during antenatal visits in several pregnancies or frequently sick children. Although this present study indicates that the respondents’ certain health knowledge improved compared to the findings of the Sierra Leone demographic and health Survey 2008 [4], however, the respondents’ knowledge on certain health parameters still remain unsatisfactory. For instance, knowledge on ORS and its use was found to be adequate with 243 (99.6%) respondents knowing ORS and 224 (94.1%) able to specify its use but only 39 (19.1%) were able to correctly explain the life saving procedure of preparing ORS from sugar, salt and water when the readymade ORS is unavailable. Knowledge on diarrhoea and its management was low with only 43.9% knowing that a child with diarrhoea need to be given more fluid; only 17.6% knew that breast milk followed by ORS if necessary is the management of choice for a child under six months old with diarrhoea; and only 11.9% of respondents were able to identify all the four preventive methods of diarrhoea included in the questionnaire. Similar findings were also found for malaria, pneumonia and HIV/AIDS with respondents admitting hearing of these conditions but lacking appropriate knowledge on their preventions and managements. Knowledge on the danger signs of pregnancy was low (not included in the Tables).

One of the aims of this study was to find out the relationship between the respondents’ health knowledge and the husband’s commitment to maternal and child health care. Fifty-nine percent of the respondents’ husbands were farmers, 11.9% were students. 31.6% of participants were not staying with their husbands, which resulted in child health care within the mother alone. Each participant’s husband had an average of 4.4 children; 38.9% of the participants’ husbands had two or more wives; only 19.7% of the participants rated their husbands’ commitment to maternal and child care as very good and only 1.2% of the interviewed women said their husbands performed all the four roles included in the questionnaire. These summaries indicated that men’s involvement in maternal and child care were not satisfactory.

This study has some limitations. In the first place, the findings of this cross sectional study on health knowledge levels, household information, treatment seeking and preventive behaviours and other results are only representative of areas selected in the Bombali district rather than the entire population of Sierra Leone. Second, the findings could be biased due to the fact that one of the clusters/villages had a regional clinic (Kabombeh), thus giving its representative participants relative advantage in terms of distance to healthcare, source of medicines and health promotion information. This made inter-cluster analyses of health knowledge less informative. Thirdly, as a direct consequence of the selected area, higher numbers of Limbas (66.0%) and Temnes (31.1%) were included in this study with little or none of the other tribes of Sierra Leone.

Despite the limitations highlighted above, the method employed in this study has some useful implications and provide information that could complement previous similar studies and future studies in this respect. Very few health knowledge, attitude and behaviour studies bring together the range of issues covered in the present study. Most studies conducted on mothers or women of reproductive age were done on single themes such as knowledge and practices on breastfeeding [54], [55], prevention and treatment or management of diarrhea [56], [57], pneumonia [58], [59], and malaria [60], [61], and as well as on knowledge and practice surrounding antenatal care and delivery [62], [63]. Of the studies mentioned above, many were conducted at the healthcare delivery point (facility level) and/or in urban settings and very few studies attempted to assess women’s health knowledge, direct household observation and the contributions of husbands to maternal and child healthcare and investigate other practices on a range of issues related to child health at the household level in rural settings. Exceptions are publications in Ghana which examined community effects such as the proportion of literate adults and the presence of a market on health knowledge in Ghana [64] and a study in Guinea that assessed the knowledge and reported practices of men and women on maternal and child health in rural Guinea Bissau [46]. As integrated approaches to the reduction of child mortality at the community level become more widespread, it becomes critical to develop tools that can capture a range of knowledge and practice of women and men at the household level in rural settings in order to plan appropriate and effective interventions [46]. In addition, unlike other studies on health knowledge in rural settings that exclusively report descriptive statistics (numbers and percentages) or simple scoring system which increases by one for each answer that is correct [65], [66], this study reported a weighted knowledge score derived by the inverse of the possible correct responses given by each respondent. Hence the authors of this study believe this method could be a useful tool in similar studies involving health knowledge in rural settings.

## Conclusions

Empowering communities with appropriate health knowledge is an appropriate and cost effective means of achieving the Millennium Development Goals especially the health-related ones (MDGs 4, 5 & 6). However, prior to formulating health promotion programmes, it is vital to assess the health knowledge of the community using specially designed surveys. Similar assessment is of immense importance in monitoring progress in already implemented programmes. Reports of this study revealed low levels of appropriate health knowledge on some of the major factors responsible for child mortality such as pneumonia, diarrhoea and malaria; as well as on breastfeeding and risk signs that dictate institutional delivery during pregnancy. It also found that the majority of births occurred at home mostly attended by Traditional Birth Attendants (TBAs) and that poor practices existed in relation to the care of the umbilical cord and immediate breastfeeding. Household sanitation and hygiene practices were found to be poor and that money for treatment and transportation still pose barriers to effective treatment seeking in rural settings. Therefore, health education activities that target these areas of knowledge and practice should be an integral part of an intervention package to improve maternal and child survival in rural Sierra Leone. However, high levels of vaccination coverage were found in this study.
